# Important roles of Vilse in dendritic architecture and synaptic plasticity

**DOI:** 10.1038/srep45646

**Published:** 2017-04-03

**Authors:** Jin-Yu Lee, Li-Jen Lee, Chih-Chen Fan, Ho-Ching Chang, Hsin-An Shih, Ming-Yuan Min, Mau-Sun Chang

**Affiliations:** 1 Institute of Biochemical Sciences, College of Life Science, National Taiwan University, Taipei, Taiwan; 2 Graduate Institute of Anatomy and Cell Biology, National Taiwan University, Taipei, Taiwan; 3 Institute of Brain and Mind Sciences National Taiwan University, Taipei, Taiwan; 4 Neurobiology and Cognitive Science Center, National Taiwan University, Taipei, Taiwan; 5 Department of Superintendent Office, Mackay Memorial Hospital, Taipei, Taiwan; 6 Department of Medical Laboratory Science and Biotechnology, Yuanpei University, Hsinchu, Taiwan; 7 Department of Medical Technology, Jen-Teh Junior College of Medicine, Nursing and Management, Miaoli, Taiwan; 8 Department of Life Science, College of Life Science, National Taiwan University, Taipei, Taiwan; 9 Institute of Biological Chemistry, Academia Sinica, Taipei, Taiwan

## Abstract

Vilse/Arhgap39 is a Rho GTPase activating protein (RhoGAP) and utilizes its WW domain to regulate Rac/Cdc42-dependent morphogenesis in Drosophila and murine hippocampal neurons. However, the function of Vilse in mammalian dendrite architecture and synaptic plasticity remained unclear. In the present study, we aimed to explore the possible role of Vilse in dendritic structure and synaptic function in the brain. Homozygous knockout of *Vilse* resulted in premature embryonic lethality in mice. Changes in dendritic complexity and spine density were noticed in hippocampal neurons of Camk2a-Cre mediated forebrain-specific Vilse knockout (Vilse^Δ/Δ^) mice. Vilse^Δ/Δ^ mice displayed impaired spatial memory in water maze and Y-maze tests. Electrical stimulation in hippocampal CA1 region revealed that the synaptic transmission and plasticity were defected in Vilse^Δ/Δ^ mice. Collectively, our results demonstrate that Vilse is essential for embryonic development and required for spatial memory.

Rho GTPases are involved in cell dynamics, cell growth, intracellular membrane trafficking, and apoptosis^1^. Among numerous identified Rho GTPases, the most known members are RhoA (Ras homologous member A), Rac1 (Ras-related C3 botulinum toxin substrate 1) and Cdc42 (cell division cycle 42). Rho GTPases switch between an active GTP-bound form and an inactive GDP-bound state. Two classes of proteins are responsible for the activation and inactivation of Rho GTPases. Guanine nucleotide exchange factors (GEFs) control GTPase activation by catalyzing the exchange of GDP for GTP^2^. By contrast, GTPase activating proteins (GAPs) increase the intrinsic GTPase activity of Rho GTPases to facilitate the hydrolysis of Rho-GTP, thereby switching them to inactive Rho GTPases^3^.

Most excitatory synapses in the mammalian brain locate at dendritic spines and the formation and dynamics of dendritic spines are mainly determined by the actin cytoskeleton^4^. Rho GTPases play important roles in the morphogenesis of dendritic spines and synaptic plasticity by modulating the actin organization^5^,^6^,^7^,^8^. RhoA is essential for stress fiber formation, whereas Rac and Cdc42 regulate the formation of lamellipodia and filopodia, respectively^9^. In particular, Cdc42 and Rac1 promote the formation and maintenance of dendritic spines, while as RhoA negatively regulates spinogenesis^10^,^11^. Morphological analysis revealed a significant decrease of spine density in the CA1 pyramidal neurons of Camk2a-cre medicated Cdc42 knockout mice^12^. Similarly, elimination of Rac1 in excitatory neurons in the forebrain *in vivo* affects spine structure and impairs synaptic plasticity in the hippocampus with defects in spatial learning^13^. In contrast, RhoA inhibition increases spine density and length and constitutively active RhoA mutant resulted in the opposite effects of RhoA ablation on dendritic spines^14^.

Vilse/Arhgap39 is a multidomain protein containing WW domain (a.a 65–95), MyTH4 (myosin tail homolog 4) domain (a.a. 760–875), and RhoGAP domain (a.a. 940–1085) for inactivating Rho GTPases^15^,^16^,^17^. Previous studies have shown that Vilse in *Drosophila* acts downstream of robo receptor through its WW domain to regulate Rac/cdc42-dependent cytoskeletal changes and neurogenesis. Genetic interactions between Vilse, slit, and robo mutants claim that Vilse transduces signals downstream of Robo receptor to regulate Rac-dependent midline repulsion in *Drosophila*^15^,^16^. In mice, Vilse interacts with connector enhancer of KSR-2 (CNK2) in the postsynaptic membrane also via WW domain to modulate Rac/Cdc42 signaling during spine morphogenesis in hippocampal neurons^17^. Structurally, MyTH4 domain has a conserved helical bundle and is often located N-terminal to a FERM domain to associate with microfilaments and microtubules^18^. However, there is no FERM domain in Vilse and the role of Vilse MyTH4 domain in regulating Rho activity and cytoskeleton remains to be elucidated.

In this report, we characterized the structural and functional phenotypes of Vilse^Δ/Δ^ mice with anatomical, physiological and behavioral means. In Vilse^Δ/Δ^ mice, the dendritic architecture in the hippocampal neurons were altered while hippocampus-mediated spatial learning/memory function was impaired. Electrophysiological recordings in the hippocampal CA1 region also revealed defects in synaptic transmission and plasticity in mutant mice. Our results led to a conclusion that Vilse is essential for embryonic development and required for dendritic structure and synaptic function.

## Results

### Generation of *Vilse* knockout mice

**Figure 1:**
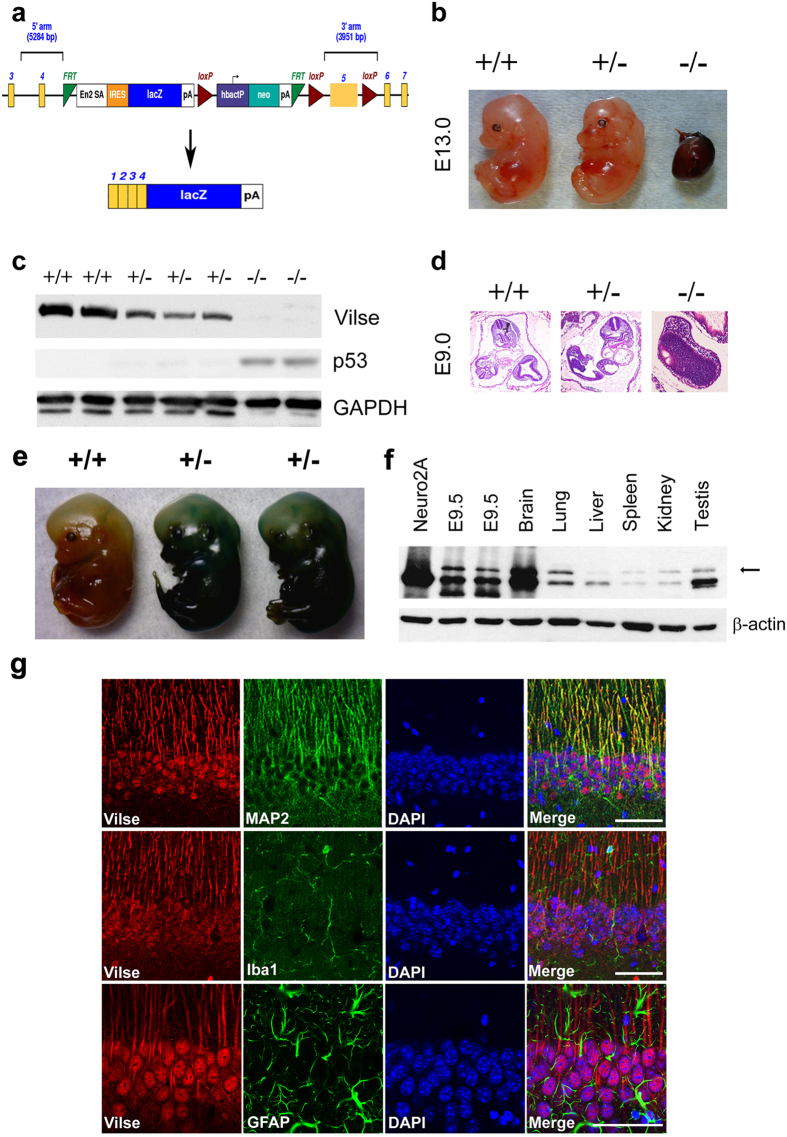
Deficiency of Vilse resulted in embryonic lethality. (**a**) Schematic representation of mouse *vilse* gene interrupted by LacZ cassette within exon 4 and 5 and two loxP sites were flanking exon 5 of *Vilse* gene. (**b**) Macroscopic inspection of embryos isolated at E13.5. ^+/+^, Wild type mice; ^+/−^, Heterozygous mice; ^−/−^, homozygous mice. All Western blots were derived from the same cell lysates, processed in identical conditions and cropped from Supplementary Fig. S3a. GAPDH was used as a loading control. (**c**) Whole embryo extracts at E10.5 were lysed in RIPA buffer for immunoblotting analysis. (**d**) Embryos were isolated from E9.0 for hematoxylin–eosin staining. (**e**) In the original targeting construct, exon 1 to exon 4 of *Vilse* are designated to be spliced to the lacZ-trapping element. Embryos at E11.5 were stained with X-gal to detect the expression of Vilse-LacZ fusion protein. (**f**) Cell extracts from E9.5 embryos, and a variety of adult tissues were immunoblotted with anti-Vilse antibody. All Western blots were processed in identical conditions and cropped from Supplementary Fig. S3b. (**g**) Double labeling of Vilse with MAP2, Iba-1, and GFAP in the hippocampal sections of wild-type mice. DAPI indicated the position of the nucleus. Bar, 50 μm.

To address the function of Vilse *in vivo*, embryonic stem (ES) cells with LacZ reporter insertion in Vilse allele were obtained from International Mouse Phenotyping Consortium (IMPC). The fragment containing exons 1 to 4 of *Vilse* is designated to be spliced to the lacZ trapping element in this targeting cassette (Fig. 1a). Chimeras capable of germ-line transmission were backcrossed to C57BL/6 mice to generate *Vilse* heterozygous mice (*Vilse*^+/−^). Male and female *Vilse*^+/−^ mice were intercrossed to produce *Vilse* homozygous mice (*Vilse*^−/−^). Of 102 progeny analyzed, *Vilse*^+/+^ and *Vilse*^+/−^ mice were approximately in a ratio of 1:2 without noticeably phenotypic differences (data not shown). However, *Vilse*^−/−^ mice died at early embryonic stages. Dead embryos could be found at E13.5 with two interrupted alleles (Fig. 1b), indicating that deficiency of *Vilse* might result in prematurely embryonic death. Whole embryo extracts were collected and immunoblotting analysis revealed the loss of Vilse protein in *Vilse*^−/−^ embryos at E10.5 (Fig. 1c). We further isolated embryos at E9.0 for H&E staining. At this stage, prospective lungs, brain, forelimb and hindlimb buds appeared in *Vilse*^+/+^ and *Vilse*^+/−^ embryos. By contrast, *Vilse*^−/−^ embryos did not display normal organogenesis, which had a severely impaired embryo morphology (Fig. 1d), indicating that the ablation of Vilse deteriorated the development and survival of *Vilse*^−/−^ embryos

Since Vilse is important for cell viability, we set out to generate Vilse’s conditional knockout mice. To determine which Cre-transgenic mice could be applied for Vilse ablation, we examined the expression of Vilse in different tissues. The exons 1–4 of Vilse are expected to be fused to β-galactosidase in the original gene-trap construct (Fig. 1a). Therefore, the Vilse-LacZ fusion protein could be visualized by the addition of X-gal as substrates. The appearance of embryos at E13.5 displayed blue colors with ubiquitously spatial patterns (Fig. 1e). Immunoblotting analysis also revealed a ubiquitous expression of Vilse in a variety of adult tissues. More interestingly, the expression of Vilse in the brain is much higher than other tissues (Fig. 1f). The double labeling experiment revealed that Vilse predominantly colocalized with MAP2 in the pyramidal cells of hippocampal CA1 neurons. By contrast, little colocalization of Vilse with Iba-1, a microglia marker, and glial fibrillary acidic protein (GFAP), an astrocyte marker, were found in the hippocampal CA1 region, indicating that Vilse mainly expressed in CA1 neurons but not in glia cells. We therefore included Camk2a-driven Cre expression strategy to generate the forebrain-specific conditional knockout of Vilse to explore the function of Vilse in the brain.

### Generation of Vilse^Δ/Δ^ mice by Camk2a-Cre mediated recombination

**Figure 2:**
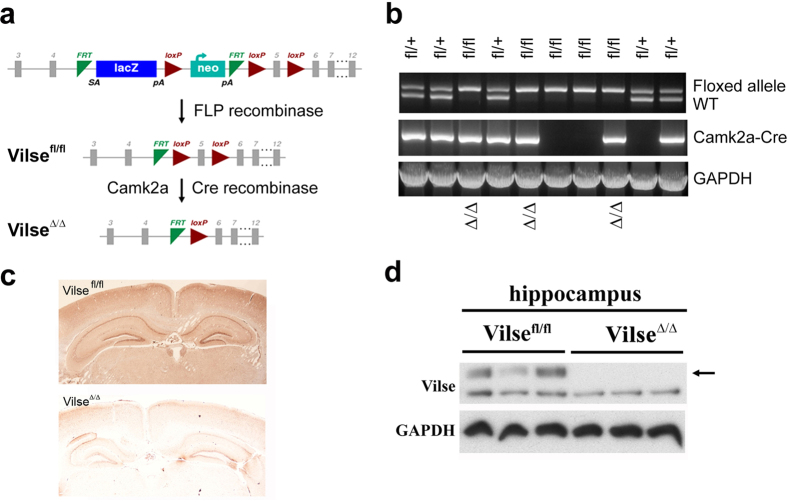
Generating conditional knockout of Vilse by deletion of exon 5 (a.a. 197–648). (**a**) Schematic model for the generation of Vilse conditional knockout mice. LacZ cassette is removed by FLP recombinase, leaving exon 5 flanked by two loxP sequences. Further removal of exon 5 is conducted by Cre recombinase. (**b**) Camk2a-iCre-Vilse^*fl/*+^ mice were mated with Vilse^*fl/fl*^ to produce Vilse^Δ/+^ and Vilse^Δ/Δ^ littermates. Upper band indicated floxed allele cleaved by Cre recombinase and lower band indicated wild-type allele. (**c**) Immunohistochemistry on Vilse^Δ/Δ^ littermates showed a reduced expression of Vilse in the forebrain area. (**d**) Immunoblot analysis of hippocampal extracts prepared from Vilse^fl/fl^ and Vilse^Δ/Δ^ littermates revealed the absence of Vilse in Vilse^Δ/Δ^ mice. All Western blots were processed in identical conditions and cropped from Supplementary Fig. S3c.

There are two *FRT* sequences flanking the gene-trap LacZ cassette. This LacZ cassette could be removed by FLP recombinase to leave exon 5 (a.a. 197–648) flanked by two loxP sequences (Fig. 2a), which can be further eliminated by Cre recombinase to produce Vilse^Δ/Δ^ mice. Therefore, *Vilse*^+/−^ mice were first mated with *FLP* transgenic mice to produce Vilse^*fl/+*^ and Vilse^*fl/fl*^ littermates. Both alleles containing lacZ cassettes were successfully removed by FLP recombinase shown as fl/fl (Fig. 2b). These Vilse^fl/fl^ pups were viable, supporting the notion that ablation of Vilse resulted in embryonic lethality. Subsequently, Vilse^fl/fl^ mice were crossed with Camk2a-Cre transgenic mice to produce Vilse^Δ/Δ^ mice. Vilse^Δ/Δ^ mice were viable with no noticeably developmental defects. Immunohistochemistry of adult Vilse^Δ/Δ^ mice confirmed a significant reduction of Vilse in the cerebral cortex and hippocampus (Fig. 2c) and immunoblotting analysis revealed the loss of Vilse in the hippocampal extracts (Fig. 2d).

### Dendritic structures of CA1 pyramidal neurons were altered in Vilse^Δ/Δ^ mice

**Figure 3:**
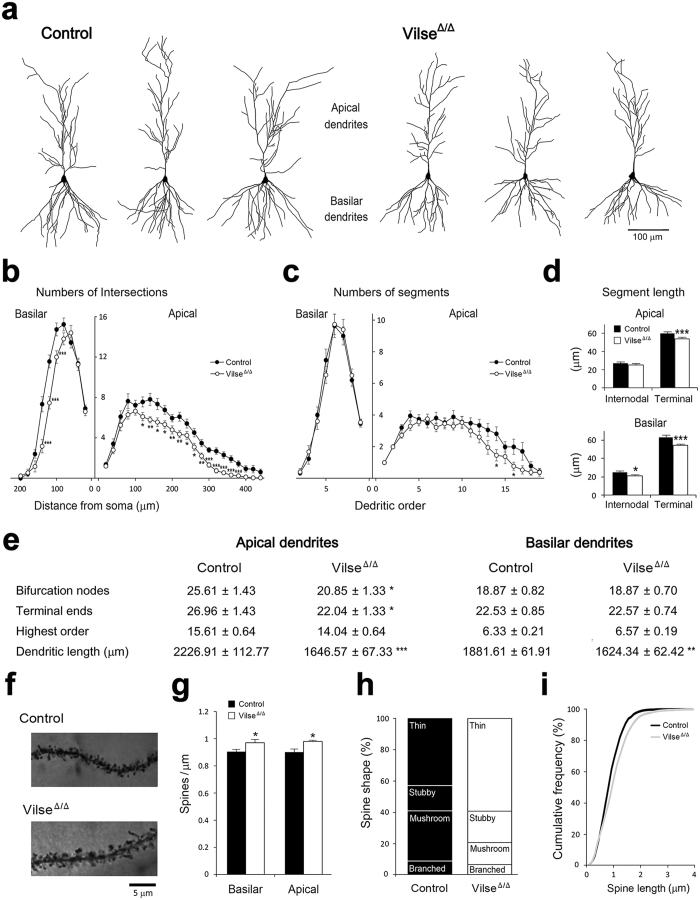
Dendritic architectures of CA1 pyramidal neurons. (**a**) Examples of reconstructed CA1 pyramidal neurons from both genotypes. (**b**,**c**) Dendritic complexity was assessed by Sholl analysis and number of segments in each dendritic order. (**d**) The length of internodal and terminal dendritic segments. Morphometric parameters of dendritic structures. (**e**) Morphometric parameters of dendritic structures. n = 30 neurons from 3 male mice for each genotype. (**f**) Examples of dendritic spines. (**g**) Density of dendritic spines. (**h**) The proportions of different spine shapes. (**i**) Cumulative frequency of spine length. Data are mean ± SEM. *p < 0.05, **p < 0.01, ***p < 0.001, two-tailed unpaired Student’s t-test.

To estimate the synaptic function in the structural aspect, we examined the dendritic architecture of CA1 neurons. Golgi-Cox impregnated CA1 pyramidal neurons (30 neurons from control and 30 neurons from Vilse^Δ/Δ^ mice) were reconstructed and analyzed using Neurolucida software (Fig. 3a). The dendritic complexity was evaluated using the concentric-ring method of Sholl in a three-dimensional manner. Compared with controls, the numbers of intersections between dendritic branches and the concentric balls were significantly reduced in both apical and basilar dendrites of CA1 neurons in Vilse^Δ/Δ^ mice (Fig. 3b). The numbers of dendritic segments (Fig. 3c), bifurcation nodes and terminal ends (Fig. 3e) were also reduced, especially in the apical dendrites of CA1 neurons in Vilse^Δ/Δ^ mice. These results indicated a reduction in dendritic complexity in mutant mice. We further analyzed the length of dendritic segments. Compared with those in controls, the length between two bifurcation nodes (intermodal length) was shorter in the basilar dendrites of CA1 neurons in mutants, while the length of terminal segments was reduced in both apical and basilar dendrites of these cells (Fig. 3d), leading to the shorter dendritic length in Vilse^Δ/Δ^ mice (Fig. 3e). These results revealed that the elongation and bifurcation of dendrites are affected in the absence of Vilse. We also characterized the density of dendritic spines, the protrusions on the dendrites where the excitatory synapses reside. Interestingly, CA1 pyramidal neurons in Vilse^Δ/Δ^ mice had greater spine density in both apical and basilar dendrites than those in control mice (Fig. 3f). We further examined the features of dendritic spines (Fig. 3g). Compared with those in control mice, greater portion of immature spine (thin spines) (Fig. 3h) and longer spine length (Fig. 3i) was noticed in the CA1 pyramidal neurons of Vilse^Δ/Δ^ mice (2094 spines counted in control mice and 2681 in Vilse^Δ/Δ^ mice). Additionally, we examined the dendritic architecture of granule cells in the dentate gyrus (DG). Similarly, reduced dendritic length and complexity and greater spine density were found in DG cells of Vilse^Δ/Δ^ mice (Supplementary Fig. 1). DG granule cells in Vilse^Δ/Δ^ mice also had more immature spines compared with those in control mice (2834 protrusions counted in control group and 2302 in Vilse^Δ/Δ^ mice, Supplementary Fig. 1g). Together, the morphological alterations of hippocampal neurons in the absence of Vilse suggested the changes of hippocampal function in Vilse^Δ/Δ^ mice.

### Spatial memory impaired in Vilse^Δ/Δ^ mice

**Figure 4:**
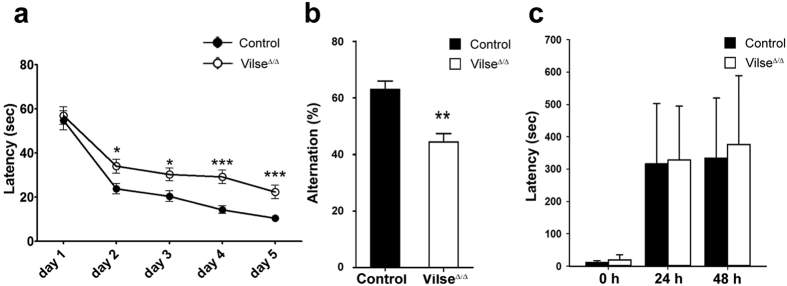
Impaired spatial memory in Vilse^Δ/Δ^ mice. (**a**) Individual mouse in control and Vilse^Δ/Δ^ groups was placed in a water maze and allowed to swim to the hidden platform four times a day for five days. The cutoff time was 90 seconds. Swimming path and time spent in quadrants of the water pool were recorded and the latency to locate the platform was averaged and analyzed in each day. *p < 0.05, **p < 0.01, ***p < 0.001 by student’s t-test. (n = 18; 6 males, 12 females). (**b**) In Y-maze test, each mouse was placed at the end of one arm and allowed to move freely through the maze during an 8-min session. Comparison of spontaneous alternation between control and Vilse^Δ/Δ^ mice in Y-maze test was performed. Columns represent the mean ± S.E.M, n = 7 mice for each genotype, **p < 0.01, unpaired t-test. (**c**) Passive avoidance test. Mice were placed into the light side of the chamber and the latency to enter the dark side was measured. A mild foot shock is delivered upon entry to the dark side of the chamber. Memory of passive avoidance was assessed at 24 h and 48 h (n = 7). n.s., not significant.

To evaluate the function of the hippocampus in Vilse^Δ/Δ^ mice, hippocampus-mediated spatial learning/memory tests were performed. In training phase of Morris water maze test, Vilse^Δ/Δ^ and control mice behaved similarly in finding the hidden platform on Day 1, suggesting the comparable visual and mobile capabilities among control and Vilse^Δ/Δ^ mice. However, during day 2 to day 5, a remarkably longer latency to find the hidden platform was exhibited in Vilse^Δ/Δ^ mice compared with control groups, suggesting a poorer spatial learning ability in Vilse^Δ/Δ^ mice (Fig. 4a). To evaluate the spatial memory of mice, a probe test was performed on day 6 in which the hidden platform was removed and swim paths of mice were traced. As anticipated, control mice swam to the removed platform areas faster than Vilse^Δ/Δ^ mice did (Supplementary Fig. S2). Male and female mice displayed similar patterns (Supplementary Fig. S2), indicating that gender differences in Vilse^Δ/Δ^ mice were not associated with impaired spatial memory. Furthermore, in the Y-maze test, Vilse^Δ/Δ^ mice also displayed a defective spatial working memory compared with control mice (Fig. 4b). However, the fear memory measured by passive avoidance test did not imply a significant difference between control and Vilse^Δ/Δ^ mice (Fig. 4c). Together, these results indicated the hippocampus-mediated spatial learning/memory function is impaired in Vilse^Δ/Δ^ mice.

### Reduced long-term potentiation in hippocampal CA1 area of Vilse^Δ/Δ^ mice

**Figure 5:**
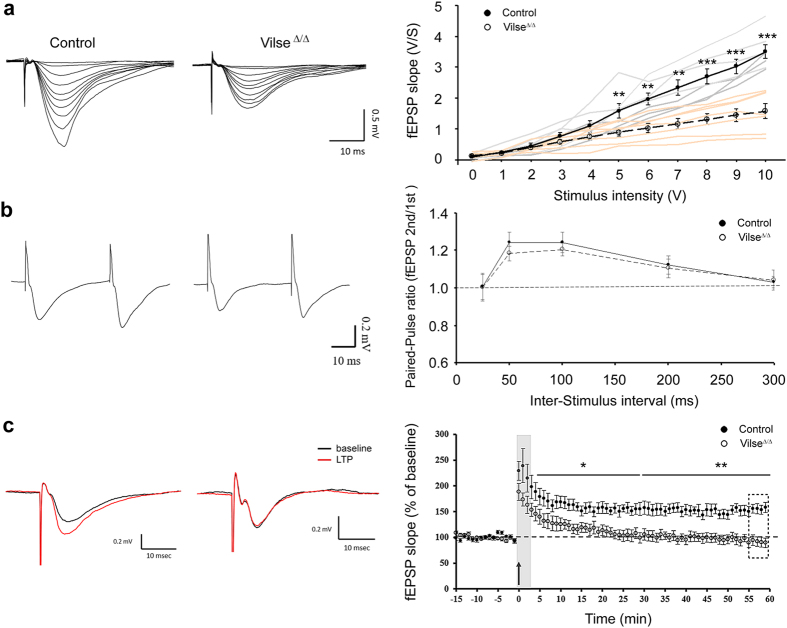
The field EPSP (fEPSP) recording at CA1 synapses in hippocampal slices. (**a**) Basal synaptic transmission was conducted and stimulus intensity was plotted against the slopes of fEPSP for 8-week-old mice (n = 7 slices from 3 control male mice; n = 7 slices from 3 male Vilse^Δ/Δ^ mice; **p < 0.01, ***p < 0.001 by one-way ANOVA). (**b**) Paired pulse facilitation. Four inter-stimulus intervals were recorded to show the percentage of paired pulse facilitation (n = 7 slices from 3 control male mice; n = 7 slices, 3 male mice from male Vilse^Δ/Δ^ mice; p = 0.62 at 50 ms, p = 0.58 at 100 ms by one-way ANOVA). (**c**) Long-term potentiation. An arrow indicated the start of high-frequency stimulation. The gray and dotted boxes represented averaged points for statistic comparison of PTP and LTP, respectively. Inserted traces shown on the top are representative recordings from control and Vilse^Δ/Δ^ slices during baseline (black trace) and 55-60 min after high-frequency stimulation (red trace) (n = 11 slices from 4 control male mice; n = 10 slices from 4 male Vilse^Δ/Δ^ mice; *p < 0.05, **p < 0.01).

To bridge the structural defects in hippocampal neurons and impaired hippocampal function in Vilse^Δ/Δ^ mice, the features of synaptic transmission in the hippocampal CA1 neurons were examined. We recorded field excitatory postsynaptic potential (fEPSP) in hippocampal CA1 slices taken from control and Vilse^Δ/Δ^ mice. Input-output curves showed that the slope of the field excitatory postsynaptic potential (fEPSP) was significantly lowered in Vilse^Δ/Δ^ mice compared with control mice as the stimulus intensity increased (5–7 V, P = 0.002; 8–10 V, P < 0.001) (Fig. 5a), reflecting a dampened basal synaptic transmission in Vilse^Δ/Δ^ mice. We next measured the responses to paired-pulse stimuli. Paired-pulse facilitation (PPF) is a character at the Schaffer collateral-CA1 synapse and considered as a form of short-term synaptic plasticity in which the second response to the closely spaced paired stimuli is increased due to residual Ca^2+^ in the presynaptic nerve terminal from the first stimulus^19^. No difference in the PPF between the control and Vilse^Δ/Δ^ mice was noticed, indicating that Vilse deficiency did not affect short-term presynaptic plasticity (Fig. 5b). Long-term potentiation (LTP), a use-dependent change in synaptic strength, has been well established as a cellular substrate for information storage in the brain^20^. We compared the properties of LTP of fEPSP in hippocampal CA1. After a period of at least 10 min baseline fEPSP recording, application of high frequency-stimulation immediately induced a post-tetanus potentiation (PTP), followed by a long-term potentiation (LTP) of fEPSP in slices. In wild-type mice, the PTP and LTP were 233 ± 27% and 161 ± 8% of the baseline, respectively. In contrast, the PTP and LTP were 175 ± 12% (p < 0.05, paired *t* test) and 91 ± 8% of the baseline (p = 0.450, paired t-test), respectively. The significant difference between control and Vilse^Δ/Δ^ mice was found in LTP (p < 0.01, Mann-Whitney *U*-test) but not in PTP (p = 0.073, Mann-Whitney *U*-test), indicating a deficit in long-term synaptic plasticity in Vilse^Δ/Δ^ mice (Fig. 5c).

## Discussion

To avoid the premature lethality, forebrain neuron-specific conditional Vilse knockout mice were generated. Camk2a-mediated Vilse ablation in mice did not result in premature embryonic death, suggesting that Vilse might not be essential for cell viability in neuronal cells. We then evaluated the consequences of Vilse knockout in the aspects of neuronal morphology and synaptic transmission in the hippocampal neurons and hippocampus-mediated spatial learning and memory function. Results from water maze and Y-maze tests revealed the defective spatial memory in Vilse^Δ/Δ^ mice. Defective spatial memory in mutant mice could be reasoned by reduced synaptic transmission and impaired synaptic plasticity such as LTP as well as the morphological alterations in dendritic complexity and spine properties in hippocampal CA1 neurons. The structural and functional changes of neurons in Vilse^Δ/Δ^ mice are likely due to the dysregulation of actin cytoskeletons resulted from the changes of Rho GTPases downstream of Vilse.

Rho GTPases not only regulate actin filament reorganization, but also participate in CTL- and Fas-induced apoptosis. Dominant-negative mutants of Rho GTPases and *Clostridium difficile* toxin B, an inhibitor of all Rho GTPases, inhibit cellular susceptibility to Cytotoxic T lymphocyte- and Fas-induced apoptosis^21^. Furthermore, Rho GTPase signaling pathways has been shown to be associated with apoptosis and the killing of superfluous cells is important during embryonic development^22^, indicating that loss of Vilse may potentiate the over-activation of Rho GTPases in embryos and consequently result in embryonic lethality. Intriguingly, Camk2a-mediated Vilse ablation in the forebrain did not result in a significant increment of RhoA GTPase activity (data not shown), suggesting that the loss of Vilse might be compensated by other RhoGAPs to modulate RhoA GTPase activity.

It is always a contention that there are approximately 70 RhoGAPs, from yeast to human, in contrast to 22 Rho proteins^3^. One plausible explanation is that some GAPs have preferential tissue expression and tissue specific functions. Their specific spatial and temporal expressions during development enable them to be tightly coped with Rho GTPases and modulate synaptic function through the interaction with diverse effectors^23^. Several GAPs have been shown to uniquely regulate synaptic development and plasticity^24^,^25^,^26^. We used forebrain-specific Vilse conditional knockout mice model to demonstrate that Vilse is involved in the modulation of dendritic architecture including dendritic length, branching property as well as the density, shape and length of dendritic spines. In CA1 and DG neurons of Vilse^Δ/Δ^ mice, greater spine density was noticed. It seemed odd to correlate this result with the reduced synaptic transmission. Since the profiles of dendritic spines are important for the transmission and integration of neural signals^27^, altered dendritic architectures in CA1 and DG cells of Vilse^Δ/Δ^ mice may result in defects of the synaptic transmission and integration of neural information. Compared with the controls, greater amounts of immature and unstable spines were observed in CA1 and DG neurons of Vilse^Δ/Δ^ mice, in line with the *in vitro* study of interfering the binding between Vilse and CNK2^17^. Removal of Vilse would affect the structural integrity of postsynaptic scaffold which might in turn influence the distribution and function of postsynaptic receptors and ion channels and result in impaired synaptic transmission. The increased density of dendritic spines could be a compensatory change. Alternatively, these excess dendritic spines might be spared from synaptic activity-mediated spine pruning. Indeed, this notion is partly supported by electrophysiological examinations on basal synaptic transmission and long term potentiation. Little change in paired pulse facilitation suggests that short-term presynaptic plasticity at CA1 synapse is not altered in Vilse^Δ/Δ^ mice. Nevertheless, the capability for CA1 synapses to undergo the use-dependent change is limited in Vilse^Δ/Δ^ mice as LTP expression is impaired. Since the hippocampus is specific to spatial memory, these observations, including dendritic architecture and electrophysiological examinations, echoed the results of water maze and Y maze tests and provide evidence that Vilse is an important player in synaptic plasticity for spatial memory. Together, the structural changes of dendritic architecture and disruption of postsynaptic integrity might account for the hippocampal LTP deficiency and behavioral deficits in hippocampus-related learning and memory tasks in Vilse conditional KO mice.

## Methods

### Antibodie

Mouse anti-Vilse monoclonal antibody was raised against 6xHis-tagged Vilse recombinant protein (a.a. 900–1114). Rabbit anti-MAP2 antibody was obtained from Millipore (Temecula, CA). Goat anti-Iba-1 antibody was from GeneTex (Hsinchu, Taiwan). Rabbit anti-GFAP antibody was from DAKO (Midland, Canada). Mouse anti-α-tubulin antibody was from Sigma-Aldrich (St. Louis, MI). (Irvine, CA).

### Whole cell extracts

Whole cell extracts were prepared with RIPA lysis buffer. To prepare cytosolic and nuclear extracts, 1 × 10^8^ cells were lysed in buffer A (10 mM HEPES (pH 7.9), 10 mM KCl, 1.5 mM MgCl_2_, 0.34 M sucrose, 10% glycerol, 1 mM DTT, 0.1% Triton X-100, protease inhibitors). After incubation at 37°C for 5 min, nuclei were lysed in 1 mL of buffer B (3 mM EDTA, 0.2 mM EGTA, 1 mM DTT, protease inhibitors). Insoluble chromatin was separated via centrifugation (5 min, 2,500 rpm) and resuspended in 0.5 mL Laemmli buffer and then sonicated for 5 min for SDS-PAGE.

### Immunofluorescence

Mice were perfused with PBS followed by paraforaformaldehyde fixation. The fixed brains were cryoprotected in 30% sucrose and cut to 30 mm thick sections. The sections were blocked in 10% normal goat serum and 2% bovine serum albumin in PBST (phosphate-buffered saline and 2% Triton X-100) and incubated with individual primary antibodies against Vilse, MAP2, Iba-1, and GFAP in blocking buffer overnight. Subsequently, DyLight 488- and Alexa fluor 594-conjugated secondary antibodies (Jackson ImmunoResearch Laboratories, West Grove, PA) were applied. Sections were coverslipped with antifade mounting medium (Southern Biotech, Birmingham, AL) and examined on a confocal microscope (Leica TCS SP5, Wetzlar, Germany).

### Generation of Vilse conditional knockout mice

All animal studies were performed in compliance with the guidelines of the Institutional Animal Use and Care Committee, Academia Sinica. An ES cell containing LacZ reporter-tagged insertion in Vilse/Arhgap39 allele were obtained from International Mouse Phenotyping Consortium (IMPC) using a combination of both a reporter-tagged and a conditional mutation^28^. Based on the original construct, exon1 to exon4 of *Vilse* is spliced to a lacZ trapping element in the targeting cassette containing the mouse En2 splice acceptor and the SV40 polyadenylation sequences^19^. This ES cell was injected into C57BL/6 blastocysts to generate chimeric mice. Germ-line transmission of the mutant allele was tested by PCR while two independent male chimeric mice were back-crossed with female C57BL/6 mice to generate Vilse heterozygous F1 offsprings. Conditional alleles were further generated by removal of the gene-trap LacZ cassette by FLP recombinase, which reverts the mutation to wild type allele, but leaving loxP sites on either side of exon 5 (a.a 197–648). Floxed homozygous male and female mice (Vilse^fl/fl^) were mated with the mouse calcium/calmodulin-dependent protein kinase II alpha promoter driving Cre recombinase transgenic mice (Camk2a-Cre mice, a gift from Dr. Che-Kun Shen, Academia Sinica) to produce Vilse^Δ/Δ^ mice. All animal studies were performed in compliance with the guidelines of the Institutional Animal Use and Care Committee, Academia Sinica.

### Genotypin

Mouse tail DNAs were isolated using EasyPure Genomic DNA spin kit (Bioman, Taipei, Taiwan) according to manufacturer’s instructions. The following pairs of primers were used, FRT1, 5′-TACTACGTGCACTCAGGTGATCCT-3′. LoxP6, 5′-AGCAGGTCACAAATGTCACTCTGC-3′. Camk2aF1, 5′-GGTTCTCCGTTTGCACTCAG-3′. iCreR1, 5′-TCCCTCACATCCTCAGGTTC-3′

FRT1 and LoxP6 were used to amplify the wild-type and floxed Vilse allele with products of 380 and 570 bp, respectively. Camk2aF1 and iCreR1 were used to amplify the presence of Camk2a-iCre with a product of 380 bp. PCR reaction was performed at 95 °C for 7 min followed by 30 cycles of 95 °C for 20 s, 53 °C for 30 s, and 72 °C for 60 s.

### Golgi-Cox impregnation and morphometric analyses

After transcardiac perfusion with phosphate-buffered saline and fixative (4% paraformaldehyde in phosphate buffer, pH 7.4), whole brains were taken and immersed in the impregnation solution from the FD Rapid Golgi Stain kit (NeuroTechnologies, Ellicott City, MD) for Golgi-Cox impregnation as previously described^29^. In brief, after 3 weeks of impregnation, brain samples were sectioned at a thickness of 150 μm and incubated with a mixture of developer and fixer solutions (FD Rapid Golgi Stain kit), washed and mounted. The pyramidal neurons in the hippocampal CA1 region and granule cells in the dentate gyrus (DG) were examined under a light microscope with a 20x objective lens for dendritic morphology and 100x objective lens for spine analysis, respectively. Series of pictures were taken by a CCD camera with the aid of computer-controlled motorized stage using the Stereo Investigator system (MBF Bioscience, Williston, VT). The morphology of selected neurons was reconstructed and analyzed with Neurolucida software (MBF Bioscience). Data were expressed as the mean ± SEM. Two-tailed unpaired Student’s t-test was used for statistical analysis.

### Morris water maze

All experiments regarding behavior tests, Golgi stain, and electrophysiology were performed in accordance with guidelines approved by National Taiwan University College of Medicine and College of Public Health (20150132). Mice were reared in the animal facility of National Taiwan University under a 12-h light/dark cycle with free access to food and water. The properties of hippocampus-mediated spatial learning and memory were evaluated using Morris water maze test^30^ with minor modifications. Briefly, the circular pool was 1.88 m in diameter and contained water (temperature 19 °C) that was made opaque with non-fat milk. A hidden platform was put in a fixed location 1 cm below the water surface. During the training period, individual mouse was placed in the water facing the pool wall at one of four start points (north, south, east, or west). Upon release into the water, the mouse was allowed to swim for 90 secs to locate the hidden platform. An additional 30 secs were then allowed for mice to stand still on the platform and escape from the water before being removed. If the mouse failed to stand on the platform, it was guided to the platform and remained there for 30 sec. Four trials of different start points were given each day for 5 consecutive days. After 5-day training period, another test trial was given in which the platform was removed. Latencies to reach the hidden platform and the swim paths were recorded with an automatic video tracking system.

### Y-maze test

Mice were placed individually at the end of one arm of the Y-maze and allowed to move within three arms freely for an 8-min period. The total number and series of arm entries were recorded. The number of non-overlapping entrance sequences (e.g. ABC, BCA) was defined as the number of alternations. The spontaneous alternation (%) was calculated as: (number of alternations)/ (total number of arm entries-2) × 100^31^.

### Passive avoidance tes

During habituation, mice were placed into the light side of the chamber and the latency to enter the dark side was recorded. On the training day, a mild foot shock is delivered upon entry to the dark side of the chamber. Memory of passive avoidance was assessed at 24 h and 48 h after training and the latency to enter the dark side was analyzed.

### Electrophysiology

Vilse^Δ/Δ^ or control mice aged 8 weeks were decapitated and their brains were quickly removed and placed in ice-cold artificial cerebrospinal fluid (ACSF) containing (in mM) NaCl, 119; KCl, 2.5; NaHCO_3_, 26.2; NaH_2_PO_4_, 1; MgSO_4_, 1.3; CaCl_2_, 2.5; glucose, 11; pH was adjusted to 7.4 by gassing with 5% CO_2_ : 95% O_2_. Transverse hippocampal slices of 450 μm thickness were cut with a vibrating tissue slicer (Dosaka microslicer, Japan) and transferred to an interface-type holding chamber at room temperature (25 °C). For extracellular field potential recording, slices were transferred to an immersion-type recording chamber, superfused with ACSF containing 0.1 mM picrotoxin at room temperature. The superfusion rate was controlled at 2 mL/min. To prevent epileptiform discharge of pyramidal neurons, a surgical cut was made at the border between areas CA1 and CA3. A glass pipette filled with 3 M NaCl was positioned at stratum radiatum of CA1 to record field excitatory postsynaptic potentials (fEPSP) evoked by a bipolar stainless steel electrode (FHC, Bowdoinham, ME) placed in the vicinity of recording pipette. To stimulate Schaffer collateral branches, a constant current pulse of 0.5 ms duration (DS3, Digitimer, UK) was delivered through the stimulating electrodes every 30 s with the intensity adjusted so that 40 ± 50% of the maximal response was elicited. Long-term potentiation (LTP) of fEPSP was induced by high-frequency-stimulation consisting of 3 trains of 100 pulses at 100 Hz with inter-train interval being 10 s. All signals were filtered at 2 kHz by a low-pass Bessel filter provided by the amplifier (Axopatch-1D, Axon Instruments, Foster City, CA) and digitized at 5 kHz using CED micro 1401 interface running Signal software provided by CED (Cambridge Electronic Design, Cambridge, UK). The initial slope of fEPSP was measured for data analysis. Synaptic responses were normalized to average values measured over a baseline period. The averaged slope of fEPSPs recorded between 0–2 and 55–60 min after high frequency stimulation was used for statistical comparisons of post-tetanus potentiation (PTP) and LTP, respectively. All data given were Mean ± SEM, and were statistically compared using either paired t-tests or one-way ANOVA. The criterion for significance was P < 0.05.

## Additional Information

**How to cite this article**: Lee, J.-Y. *et al*. Important roles of Vilse in dendritic architecture and synaptic plasticity. *Sci. Rep.* **7**, 45646; doi: 10.1038/srep45646 (2017).

**Publisher's note:** Springer Nature remains neutral with regard to jurisdictional claims in published maps and institutional affiliations.

## Supplementary Material

Supplementary Information
