# Supplementary material for: Important roles of Vilse in dendritic architecture and synaptic plasticity
Source: Sci Rep. 2017 Apr 3;7:45646. doi: 10.1038/srep45646 (PMC5377306; doi:10.1038/srep45646)
Supplement: Supplementary Information [file srep45646-s1.doc]

**Supplementary Information**

**Title**

Important roles of Vilse in dendritic architecture and synaptic plasticity

**Authors**

Jin-Yu Lee, Li-Jen Lee, Chih-Chen Fan, Ho-Ching Chang, Hsin-An Shih, Ming-Yuan Min, and Mau-Sun Chang


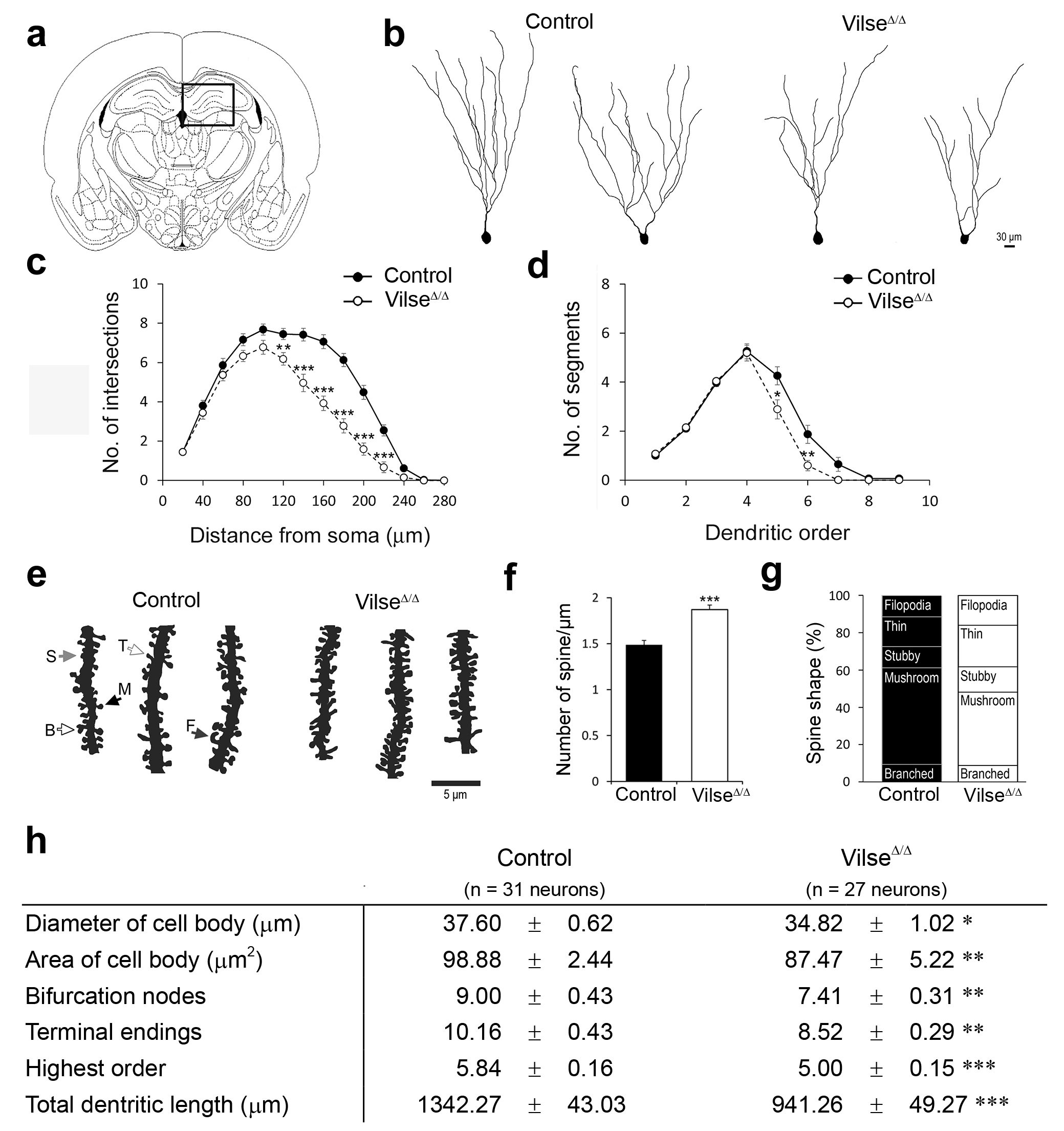


**Supplementary Figure S1.** Dendritic architectures of dentate gyrus cells in Vilse^Δ/Δ^ mice. (a) The DG of the dorsal hippocampus is indicated by the rectangle frame. (b) DG granule cells from both genotypes were collected and reconstructed. (c & d) Dendritic complexity was assessed by Sholl analysis and number of segments in each dendritic order. (e) On the dendritic segments, dendritic spines of filopodia (F), thin (T), stubby (S), mushroom (M) and branched (B) are indicated. (f) The density of dendritic spines in total was quantified. (g) The proportions of different spine shapes were estimated. *H,* Morphometric features of DG granule cells in Vilse^Δ/Δ^ and control mice. Data are mean ± SEM (* *p* < 0.05, ** *p* < 0.01, *** *p* < 0.001).


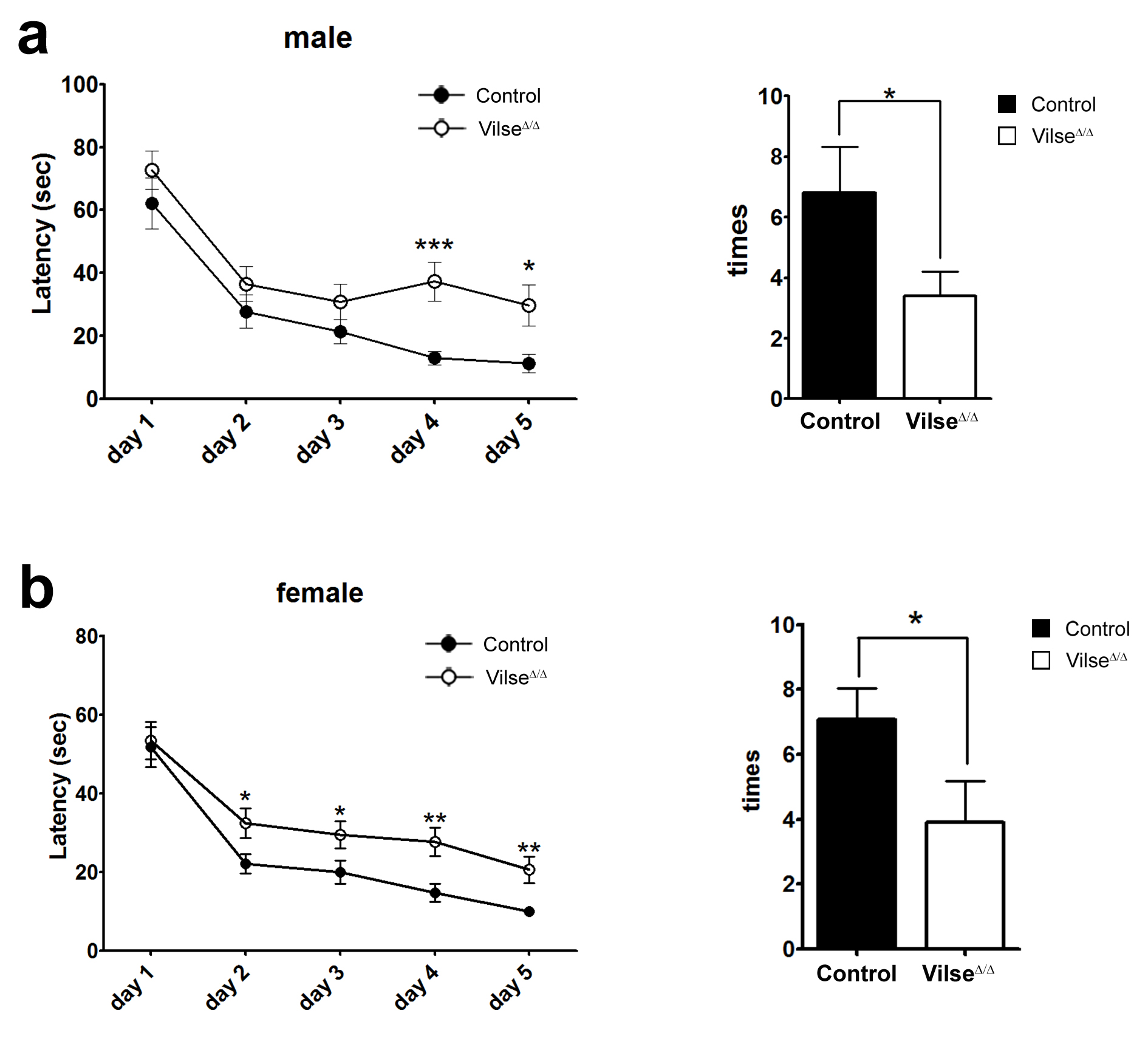


**Supplementary Figure S2.** Spatial memory impaired both in Vilse^Δ/Δ^ male and female mice. (a) Control and Vilse^Δ/Δ^ male mice were placed in Morris water maze and allowed to swim to the hidden platform. For the probe trial on Day 6, the hidden platform was removed and the number of times to the empty area in 90 sec were recorded and analyzed (right panel). * *p* < 0.05, ** *p* < 0.01, *** *p* < 0.001 by student’s t-test. n=6. (b) Control and Vilse^Δ/Δ^ female mice were placed in Morris water maze and allowed to swim to the hidden platform. For the probe trial on Day 6, the hidden platform was removed and the number of times to the empty area in 90 sec were recorded and analyzed (right panel). * *p* < 0.05, ** *p* < 0.01, *** *p* < 0.001 by student’s t-test. n=12.


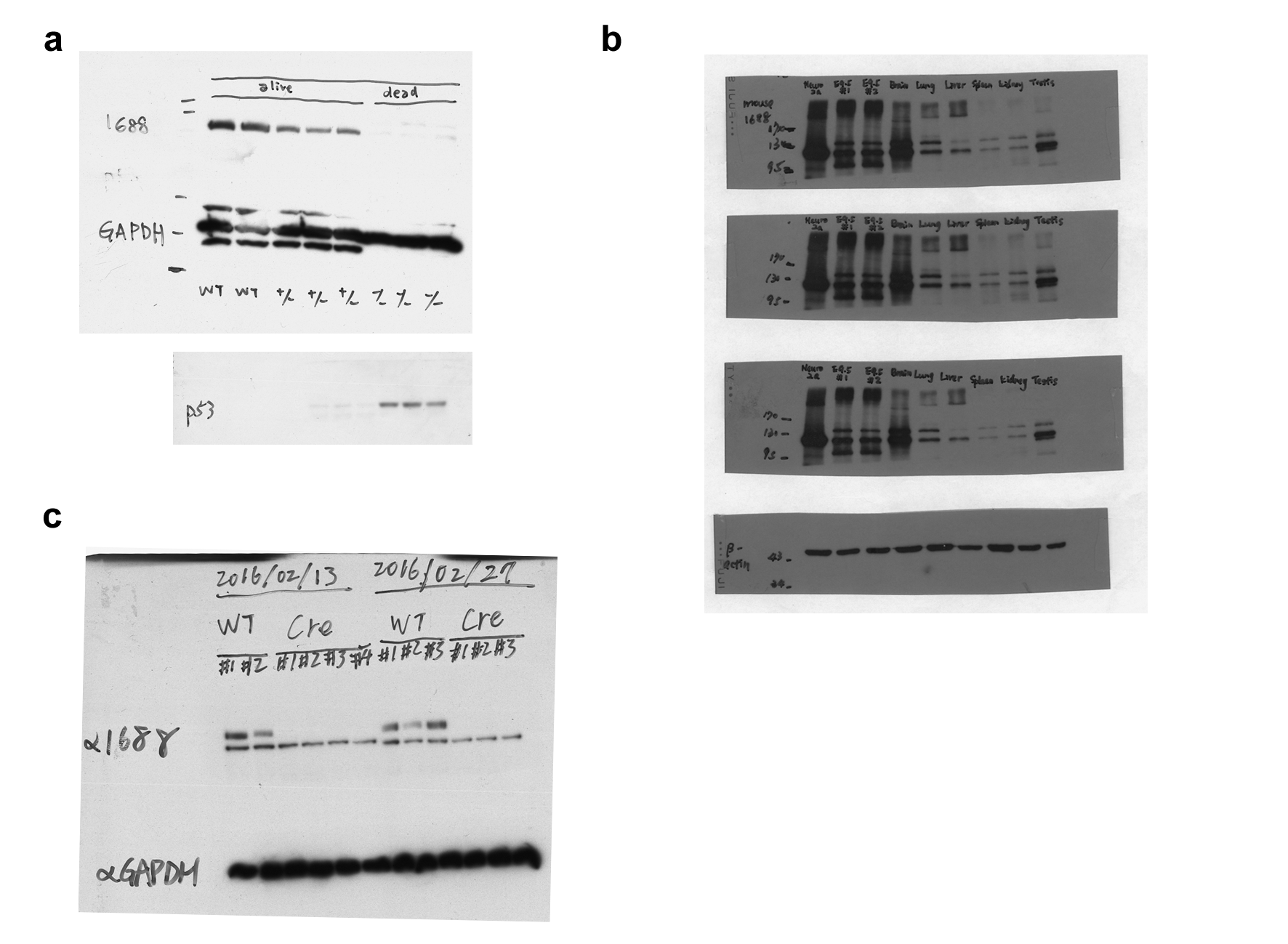


**Supplementary Figure S3.** Uncropped images for all gels and Western blots.
